# Lignocellulose@ Activated Clay Nanocomposite with Hierarchical Nanostructure Enhancing the Removal of Aqueous Zn(II)

**DOI:** 10.3390/polym11101710

**Published:** 2019-10-18

**Authors:** Xiaotao Zhang, Yinan Hao, Zhangjing Chen, Yuhong An, Wanqi Zhang, Ximing Wang

**Affiliations:** 1 College of Science, Inner Mongolia Agricultural University, Hohhot 010018, China; lianzixiaotao@163.com; 2 College of Material Science and Art Design, Inner Mongolia Agricultural University, Hohhot 010018, China; Imau_chem@163.com (Y.H.); 15034999229@163.com (Y.A.); 15049800999@163.com (W.Z.); 3 Department of Sustainable Biomaterials Virginia Tech University, Blacksburg, VA 24061, USA; 15034999339@163.com

**Keywords:** lignocellulose@ activated clay (Ln@AC), nanocomposite, Zn(II), adsorption, desorption

## Abstract

A lignocellulose@ activated clay (Ln@AC) nanocomposite with a hierarchical nanostructure was successfully synthesized by the chemical intercalation reaction and applied in the removal of Zn(II) from an aqueous solution. Ln@AC was characterized by N_2_ adsorption/desorption isotherms and X-Ray Diffraction (XRD), scanning Electron Microscope (SEM), transmission Electron Microscopy (TEM) and Fourier Transform Infrared Spectroscopy (FTIR) analysis, and the results indicate that an intercalated–exfoliated hierarchical nanostructure was formed. The effects of different adsorption parameters on the Zn(II) removal rate (weight ratio of Ln to AC, Ln@AC dosage, initial Zn(II) concentration, pH value, adsorption temperature, and time) were investigated in detail. The equilibrium adsorption capacity reached 315.9 mg/g under optimal conditions (i.e., the weight ratio of Ln to AC of 3:1, Ln@AC dosage of 1 g/L, initial Zn(II) concentration of 600 mg/L, pH value of 6.8, adsorption temperature of 65 °C, and adsorption time of 50 min). The adsorption process was described by the pseudo-second-order kinetic model, Langmuir isotherm model, and the Elovich model. Moreover, Zn(II) could be easily eluted by HCl, and the effects of HCl concentration, desorption temperature, and ultrasonic desorption time on desorbed amount were tested. Desorption studies revealed that with an HCl concentration of 0.25 mol/L, desorption temperature of 70 °C, and ultrasonic desorption time of 20 min, the maximum desorption capacity and efficiency were achieved at 202.5 mg/g and 64.10%, respectively. Regeneration experimental results indicated that the Ln@AC exhibited a certain recyclable regeneration performance. Due to such outstanding features, the novel Ln@AC nanocomposite proved to have great adsorption potential for Zn(II) removal from wastewater, and exhibited an extremely significant amount of adsorbed Zn(II) when compared to conventional adsorbents.

## 1. Introduction

Heavy metal contamination of water is a widespread environmental issue in recent years. Heavy metals seriously threaten the ecological system as well as human health due to their high mobility in water and easy bioaccumulation in living tissues through the food chain [1,2]. Among the harmful heavy metals, zinc, which is recognized as one of the most hazardous elements, is not biodegradable, even at low concentrations. Thus, zinc removal has been an important but challenging area of wastewater treatment [3,4,5]. Up to now, the applicability of various methods has been assessed for the removal of Zn(II) from effluents including ion exchange, precipitation, filtration, reverse osmosis, electrolysis, and so on [6,7,8]. In particular, adsorption is now progressing through research intended for the development of materials as adsorbents that are relatively low cost, highly efficient, flexible in design, biodegradable, simple in operation, and eco-friendly. A series of adsorbents have been widely used in Zn(II) removal from aqueous effluents [9,10,11].

Natural polymeric biomaterials are appealing for application as adsorbents due to their high adsorption capacity, availability, reusability, and nontoxic nature. Lignocellulose (Ln), the most abundant renewable polysaccharide (cellulose, hemicellulose) and aromatic polymer (lignin) on Earth, is a constituent of a variety of materials such as sawdust, sugarcane bagasse, grains, straws, stalks, leaves, and peels from cereals, among others. Its molecular chains contain a large number of active oxygen groups (–OH, –COO, –C=O, C–O–C, etc.), which are known to adsorb heavy metals. However, its poor solubility in water, weak mechanical properties, and low gravity make it inconvenient to use directly. Thus, several attempts have been made to develop more effective modified Ln adsorbents [12]. Natural layered silicates or activated clay (AC) are commonly accepted as appropriate low-cost adsorbents for their high surface area, high cation-exchange capacity, fine hierarchical structure, and expandable interlayers. However, AC adsorbs heavy metal ions only on external residual broken bonds on its surface in small amounts and has little or no affinity with organic polymers. Consequently, in order to improve its adsorption capacity, dispersive suspendability, and expandability, modifying AC has been considered a priority [13,14].

With the rapid development of nanotechnology, it has been realized that the adsorption efficiency of modified natural phyllosilicates can be prominently enhanced by introducing nanoscale organic polymer fillers [15]. To our knowledge, among the many kinds of polymer/layered silicate composites, the lignocellulose@ activated clay (Ln@AC) nanocomposite has rarely been covered with regard to the adsorption of Zn(II) from wastewater. Specifically, biopolymer Ln-based and hierarchically structured AC nanocomposite are attractive as adsorbents due to their potential mechanical properties and physicochemical characteristics as well as better chelation–complexation effects toward Zn(II) ions.

Our present work mainly focuses on the synthesis, characterization, and application of Ln@AC nanocomposite in Zn(II) removal from aqueous solutions. The Ln@AC production process is novel because it adopts the national invention patent method in China [16]. The properties of Ln@AC were investigated by analyzing the N_2_ adsorption/desorption, x-ray diffraction (XRD), scanning electron microscopy (SEM), transmission electron microscopy (TEM), and Fourier-transform infrared spectroscopy (FTIR) results. Furthermore, the adsorption and desorption capacities of Zn(II) are discussed in detail. Each factor influencing the adsorption and desorption behavior of Ln@AC including the weight ratio of Ln to AC, Ln@AC dosage, initial Zn(II) concentration, pH value, adsorption temperature, adsorption time, HCl concentration, desorption temperature, and ultrasonic desorption time was systematically studied using various scenarios. Additionally, the adsorption kinetics and isotherms with respect to Ln@AC were studied and the mechanism of Zn(II) adsorption is discussed.

## 2. Experiments

### 2.1. Materials

Ln was acquired from Beijing Huaduo Biotech Ltd., Beijing, China. The cationic exchange capacity (CEC) of purified AC (Shandong Ruicheng Chemical Co., Zibo, China) is 1 meq/g. Prior to use, the AC sample was washed with deionized water, soaked in hydrochloric acid solution for 30 min, and ground and sieved to 200 mesh size (model Φ200) for experimental use. A stock solution of Zn(II) (1000 mg/L) was prepared using analytical reagent (AR) grade Zn(NO_3_)_2_ (Tianjin Beilian Fine Chemicals Co. Ltd., Tianjin, China). Other reagents used were of analytical grade without further purification. All solutions were prepared with deionized water.

### 2.2. Ln@AC Preparation

AC was washed to neutrality using deionized water and dried at 80 °C for 24 h in a hot-air oven (Memmert VO400, Schwabach, Germany). Subsequently, AC (1.0000 g; BS210S, Sartorius, Gottingen, Germany) was swelled by deionized water (weight of AC (g): volume of water (mL) = 1:30) and stirred continuously at 60 °C for 30 min. Then, Ln (3.0000 g) was dissolved in 15% NaOH solution (weight of Ln (g):volume of NaOH (mL) = 1:30) in batches, after being magnetically stirred at 60 °C for 30 min, forming a uniform suspension [16]. Next, the Ln–NaOH suspension was slowly dropped into the AC mixture, followed by stirring at 600 rpm for 30 min. Afterward, the reaction mixture was mechanically stirred at 55 °C for 5 h. The mixture was filtered and washed several times with deionized water until the pH of the supernatant reached 7.0. After that, Ln@AC was vacuum-dried at 60 °C (DZF-6210, Shanghai, China) for 120 min until the weight was stable. All samples as adsorbents were ground and sieved through a 200-mesh sieve (model Φ200).

### 2.3. Adsorption Studies

All batch experiments were performed in a thermostatic shaker (SHA-C, Jiangsu, China) at 150 rpm. An amount of Ln@AC was accurately weighed and added to 50 mL of Zn(II) with a known concentration. The pH of the suspension was adjusted using a certain amount of NaAc–HAc buffer solution with a pH meter (PB-10, Shanghai, China). When the adsorption equilibrium was reached, the sample was withdrawn from the shaker and centrifuged at 5000 rpm (H2050R, Hunan, China) for 10 min. The upper fluid was then taken to determine the residual concentration of Zn(II) by xylenol orange spectrophotometry. The Zn(II) complex, which was added to the xylenol orange solution by adjusting the pH to 5.60 using NaAc–HAc buffer solution, was ready for use after 10 min (but no later than 30 min), and was transferred into 1 cm of quartz color dishes and scanned for absorption in the double-beam ultraviolet (UV) visible spectrophotometer (TU-1901, Beijing, China) at a wavelength corresponding to maximum absorbance of about 570 nm. Spectroscopic grade standards were periodically checked during the experiment and used to calibrate the instrument. Then, the concentrations of the samples were determined by using the linear regression equation (y = 0.3297x + 0.401, *R*^2^ = 0.9998) for Zn(II) ions. The adsorption experiments were conducted with different weight ratios of Ln to AC, Ln@AC dosages, initial Zn(II) concentrations, pH values, adsorption temperatures, and adsorption times. To consider experimental errors, three experiments were run in parallel under the same conditions, and the obtained results were based on average values. The adsorbed amount was calculated [17] by the following (Equation (1)):

(1) $$q_{t,1}=\frac{(C_{0}-C_{t,1})V_{1}}{m_{1}}$$

where *q*_t,1_ (mg/g) is the adsorbed amount of Zn(II) at time *t* (min); *C*_0_ and *C*_t,1_ (mg/L) refer to the initial and final concentration of Zn(II) at time *t* (min), respectively; *V*_1_ (L) refers to the volume of Zn(II) solution; and *m*_1_ (g) is the mass of the adsorbent. In the calculation of *q*_t,1_, no loss of Zn(II) to any other mechanism (e.g., volatilization, sorption on glassware, degradation) was assumed.

### 2.4. Desorption and Regeneration Studies

A sample (0.1000 g) of Zn(II)-loaded Ln@AC nanocomposite was accurately weighed and transferred into 50 mL of HCl aqueous solution with different concentrations. Each mixture was placed in an ultrasonic cleaning machine (KS-300EI, Ninbo, China). When the desorption equilibrium was reached, the suspension was centrifuged and the desorbed Zn(II) concentration was determined as previously mentioned. To consider the experimental errors, three experiments were performed, and the reproducibility of the results was within ±3%. The desorption capacity and desorption efficiency of the Zn(II)-loaded Ln@AC were calculated according to Equations (2) and (3) [18,19]:

(2) $$q_{t,2}=\frac{C_{t,2}\times V_{2}}{m_{2}}$$

(3) $$Desorption(\%)=\frac{q_{t,2}}{q_{\max}}\times 100\%$$

where *q*_t,2_ (mg/g) refers to the desorbed amount at time *t* (min); *C*_t,2_ (mg/L) is the Zn(II) concentration in the desorption solution at time *t* (min); *V*_2_ (L) refers to the volume of the desorption solution; *m*_2_ (g) is the mass of the Zn(II)-loaded Ln@AC; and *q*_max_ (mg/g) refers to the maximum adsorbed amount.

Repeated batch experiments were performed to examine the reusability of Ln@AC for Zn(II). After the desorption equilibrium was completed, the suspension was separated from the adsorbent by centrifugation at 5000 rpm for 10 min, washed with deionized water to neutral pH, and vacuum-dried in an oven (DZF-6210, Shanghai, China) at 55 °C for the next adsorption experiment. The adsorption and desorption data were determined and analyzed. The consecutive adsorption/desorption cyclic process was performed six times.

### 2.5. Characterization

The specific surface area and porous system of the Ln@AC were characterized based on N_2_ adsorption/desorption isotherms (ASAP 2020, Micrometrics, Norcross, GA, USA). The zeta potential of the Ln@AC nanocomposite was measured on a Zetasizer Nano (ZEN3600, Malvern Panalytical, Kassel, UK). X-ray diffraction (XRD) analysis of the powdered samples was performed using an X-ray power diffractometer with a Cu anode (Panalytical X′pert PRO, Almelo, The Netherlands) running at 40 kV and 30 mA, scanning from 4° to 18° at 3°/min. Micrographs of Ln@AC were recorded using a scanning electron microscope (SEM) (Hitachi S-4800, Tokyo, Japan). Energy dispersive X-ray spectroscopy (EDX) analysis was also performed (Hitachi S-4800, Tokyo, Japan). Before SEM observation, all samples were fixed on aluminum stubs and coated with gold. Transmission electron microcopy (TEM) images of the samples were acquired using a TEM (JEM-2010, Tokyo, Japan) at 200 kV. FTIR spectra of the Ln@AC were characterized in KBr pellets using a Fourier-transform infrared (FTIR) spectrometer (Thermo Nicolet Nexus; Thermo Fisher Scientific, Waltham, MA, USA).

## 3. Results and Discussion

### 3.1. Properties of the Ln@AC (lignocellulose@activated clay) Nanocomposite

The porosity characteristics of the studied Ln@AC from its N_2_ adsorption/desorption isotherms are listed in Table 1. It can be seen from Table 1 that the highest *S*_BET_ and *V*_tot_ for Ln@AC were 611.23 m^2^/g and 3.645 cm^3^/g, respectively, which were calculated using the t-plot method. The results indicate that compared to the AC, the porous structure of the Ln@AC was well developed, the *V*_meso_ highly increased, and the *V*_m__ac_ decreased after Ln was intercalated into the interlayer space of the AC. In addition, the structure of Ln@AC was found to be loose, with many mesopores and micropores generated on its surface, and the mesopore structure can be supported by its average pore diameter (D*_p_* = 54.09 nm). The zeta potential of the Ln@AC nanocomposite was negatively charged (−30.17 mV) and lower than the zeta potential of AC (−17.69 mV), which led to electrostatic interactions between the Ln@AC and Zn(II) ions.

**Figure 1:**
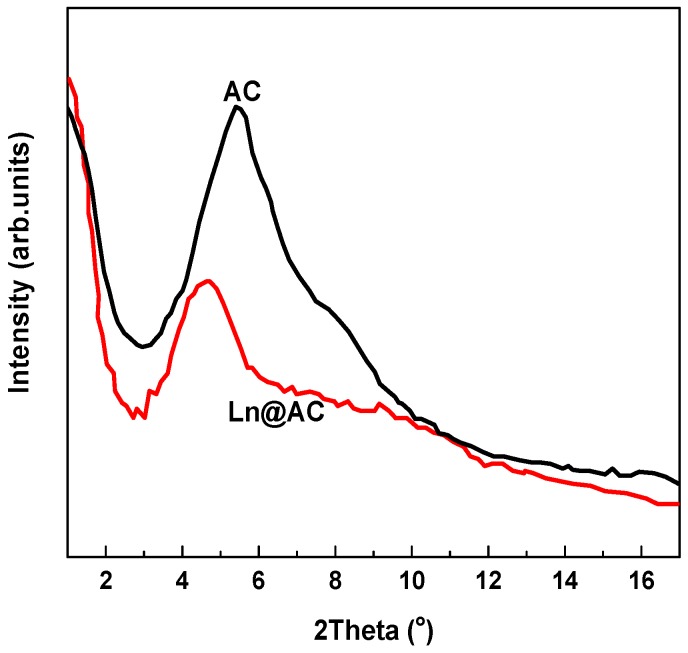
XRD patterns of the AC (activated clay) and Ln@AC (lignocellulose@activated clay) nanocomposite.

XRD is an effective method for investigating the existence of intercalation in AC. Figure 1 shows the XRD patterns of AC and the prepared Ln@AC. We found that a typical peak of AC was near 2θ = 5.40°, responding to a basal spacing of 1.63 nm, which presented typical hierarchical nanostructure features. In contrast, after intercalation with Ln, this diffraction peak significantly decreased and shifted to a lower angle (2θ = 4.52°), which was assigned to the interlayer platelet placing of 19.12 nm of Ln@AC. The magnitude of the change in peaks suggests that AC exfoliated and Ln intercalated into AC interlayers [20]. This observation indicated the formation of an intercalated–exfoliated hierarchical nanostructure in the Ln@AC nanocomposite.

**Figure 2:**
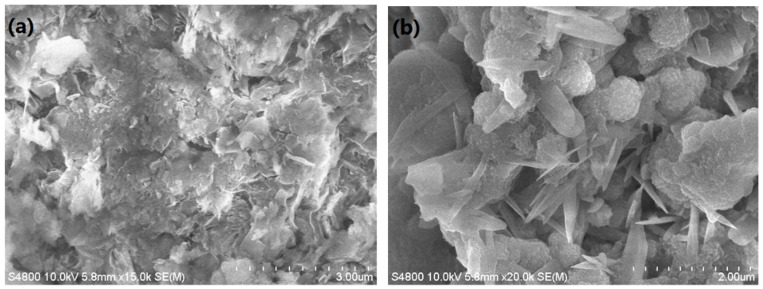
SEM (scanning electron microscopy) images of the (**a**) purified AC and (**b**) Ln@AC nanocomposite.

Micrographs of the purified AC and Ln@AC nanocomposite are shown in Figure 2. It can be seen from that the AC consisted of small particles, thin sheets, layers, and a nonporous surface (Figure 2a). However, the introduction of Ln led to a well-developed coarse porous surface with irregular pores and breaks (Figure 2b). Moreover, compared to AC, the Ln@AC nanocomposite had more micropores and mesopores, indicating that the incorporation of Ln and AC can form numerous cavities and a relatively loose surface, and this intercalated–exfoliated hierarchical nanostructure results in the increased adsorption ability of Zn(II).

**Figure 3:**
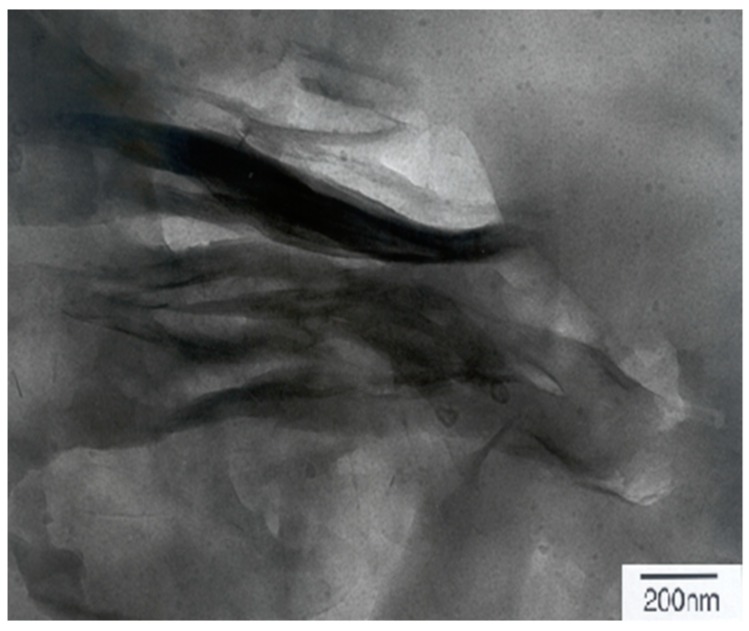
TEM (transmission Electron Microscopy) image of the Ln@AC nanocomposite.

More direct morphological evidence of the Ln@AC nanocomposite was recorded by TEM. The combination of XRD patterns and TEM analysis is a powerful method when characterizing the hierarchical nanostructure of polymer/silicate clay nanocomposites. Figure 3 shows a TEM micrograph of Ln@AC. It was clear that the thin, dispersive, and dark lines were the intersections of AC sheets, while nanoplatelets and spaces between the dark lines were Ln chain molecules. Almost all of the Ln was embedded into the interlayer, expanding the hierarchical space of the AC. Organic polymers of Ln were well dispersed within the interlayers. This confirmed that the disordered intercalated–exfoliated hierarchical nanostructure of the AC still existed in the Ln@AC nanocomposite.

**Figure 4:**
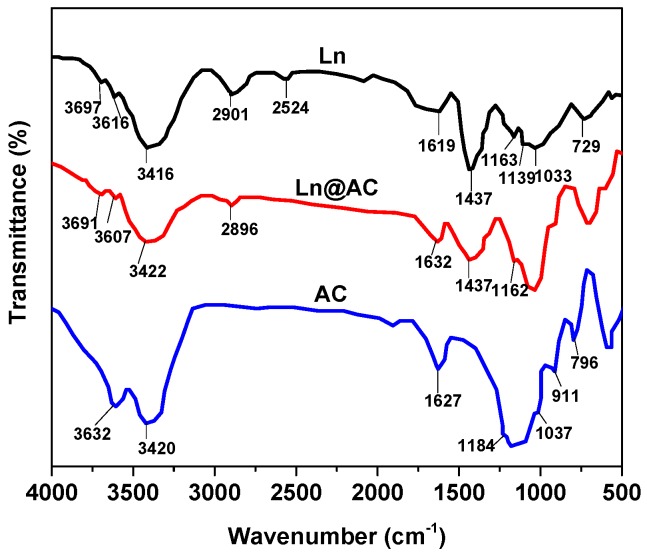
FTIR (Fourier Transform Infrared Spectroscopy) spectra of the Ln, Ln@AC nanocomposite, and AC.

The FTIR spectra of Ln, Ln@AC nanocomposite, and the AC are shown in Figure 4. Compared with the FTIR spectra of AC, the characteristic absorption band at 3632 cm^−1^, corresponding to the –OH stretching vibration of AC, almost disappeared on the spectra of Ln@AC. The adsorption band at 3420 cm^−1^, assigned to the –OH stretching vibration of H_2_O of AC and intermolecular hydrogen bonds, widened and shifted to a higher wavenumber (3422 cm^−1^) in Ln@AC. The characteristic adsorption band of Ln at 2901 cm^−1^ weakened, attributed to methyl groups of Ln. The adsorption band at 1627 cm^−1^, attributed to the characteristic absorption vibration of –COOH, weakened and shifted to a higher wavenumber (1632 cm^−1^) in the Ln@AC. The adsorption band at 1037 cm^−1^, assigned to the Si–O stretching vibration of the AC, disappeared on the FTIR spectra of Ln@AC. In addition, the adsorption bands at 1163, 1139, and 1033 cm^−1^, corresponding to the C–O–C and C–O stretching vibration of Ln, obviously weakened on the FTIR spectra of Ln@AC. The adsorption bands at 911 and 796 cm^−1^, attributed to the Al–O and Mg–O stretching vibration of AC, respectively, also weakened on the FTIR spectra of Ln@AC [21]. It can be concluded that the –OH, Si–O, and Al–O groups on the surface of the AC interact with the C–O–C, –C=O, and C–O groups in Ln through chemical complexation and coordination. The information observed from the FTIR spectra indicates that Ln polymer molecules could influence the chemical environment of the AC and may enhance the adsorption performance of the Ln@AC nanocomposite.

### 3.2. Influencing Factors on Zn(II) Adsorption

#### 3.2.1. Effect of Weight Ratio of Ln (Lignocellulose) to AC (Activated Clay)

**Figure 5:**
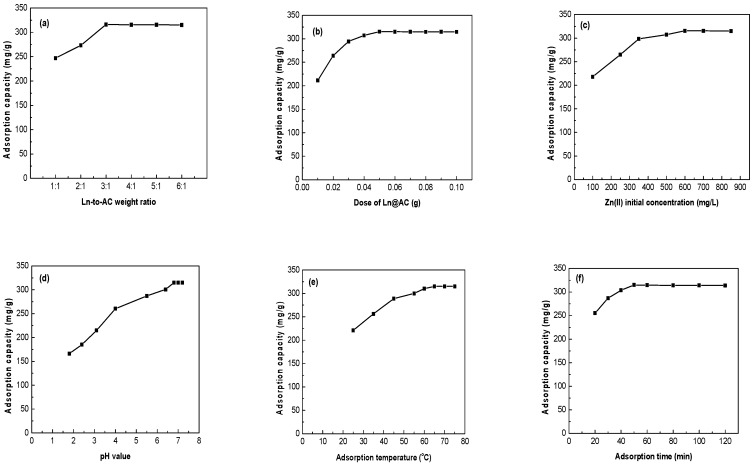
Various influencing factors on Zn(II) adsorption by the Ln@AC nanocomposite. (**a**) Effect of weight ratio of Ln to AC (weight ratio: 1:1–6:1; dosage: 1 g/L; Zn(II) concentration: 600 mg/L; pH: 6.8; temperature: 65 °C; time: 50 min). (**b**) Effect of Ln@AC dosage (weight ratio: 3:1; dosage: 0.01–0.10 g; Zn(II) concentration: 600 mg/L; pH: 6.8; temperature: 65 °C; time: 50 min). (**c**) Effect of Zn(II) concentration (weight ratio: 3:1; dosage: 1 g/L; Zn(II) concentration: 100–850 mg/L; pH: 6.8; temperature: 65 °C; time: 50 min). (**d**) Effect of pH value (weight ratio: 3:1; dosage: 1 g/L; Zn(II) concentration: 600 mg/L; pH range: 1.8–7.2; temperature: 65 °C; time: 50 min). (**e**) Effect of adsorption temperature (weight ratio: 3:1; dosage: 1 g/L; Zn(II) concentration: 600 mg/L; pH: 6.8; temperature: 25–75 °C; time: 50 min). (**f**) Effect of adsorption time (weight ratio: 3:1; dosage: 1 g/L; Zn(II) concentration: 600 mg/L; pH: 6.8; temperature: 65 °C; time: 20–120 min).

As the weight ratio of Ln to AC increased, the Ln@AC nanocomposite was easy to shrink and the AC to agglomerate, which could improve the adsorption capacity. This can facilitate the separation of Ln@AC from an aqueous solution, especially for practical operations. Figure 5a shows the effects of the weight ratio of Ln to AC on adsorption capacity. It can be seen that the adsorption capacity of Zn(II) increased with increased weight ratio, but almost kept stable when the ratio exceeded 3:1. This could be because increasing the amount of Ln is helpful to balance the initial negative charges of AC and strengthen the adsorption capacity of Zn(II). Therefore, the Ln@AC with a weight ratio of Ln to AC of 3:1 was selected in this work.

#### 3.2.2. Effect of Adsorbent Dosage

The effects of different Ln@AC nanocomposite dosages for Zn(II) removal were investigated, and the results are shown in Figure 5b. The amount of Ln@AC was changed from 0.01 to 0.10 g, while the variables initial Zn(II) concentration, pH value, adsorption temperature, and adsorption time were kept constant. As can be seen from Figure 5b, the adsorption capacity of the Ln@AC increased rapidly from 211.4 to 315.2 mg/g in Zn(II) aqueous solution. This was due to the greater availability of active sites. Beyond 1 g/L of Ln@AC, the adsorption capacity of Zn(II) ions remained steady [22]. Thus, a dose of 1 g/L Ln@AC was selected for subsequent adsorption experiments.

#### 3.2.3. Effect of Initial Zn(II) Concentration

Initial Zn(II) concentration is an important factor affecting the adsorption capacity of the Ln@AC nanocomposite. Figure 5c shows the adsorption capacity of the Ln@AC at different Zn(II) concentrations. It is clear that the adsorption capacity trend of the Ln@AC toward Zn(II) first increased sharply from 217.9 to 315.6 mg/g with increased Zn(II) concentration from 100 to 600 mg/L, then remained nearly stable with further increased Zn(II) concentration. This may be attributed to the fact that the aggregation of Zn(II) makes it almost impossible for it to diffuse deeper into the hierarchical nanostructure of the Ln@AC. Therefore, Zn(II) with an initial concentration of 600 mg/L was chosen as the ideal condition.

#### 3.2.4. Effect of pH Value

Generally, the adsorption capacity of the adsorbent is highly dependent on the pH value. The effect of the pH of the Zn(II) solution on adsorption capacity was investigated, as shown in Figure 5d. The results indicate that when the pH of the Zn(II) solution was raised from 1.8 to 6.8, the adsorption capacity increased from 166.1 to 315.1 mg/g. This can be explained by the competition between H^+^ and Zn(II) for activated sites on the Ln@AC surface at low pH levels. When the pH increased, the covered H^+^ left the Ln@AC surface, making more adsorption sites available for Zn(II) [23]. However, with a pH higher than 6.8, Zn(II) could react with more hydroxyls, resulting in facile complexation or precipitation, therefore the adsorbed amount of Zn(II) remained almost constant. It was determined that the optimum pH value for adsorption was 6.8.

#### 3.2.5. Effect of Adsorption Temperature

The relationship between temperature and adsorption capacity of the Ln@AC is shown in Figure 5e. As can be seen, the adsorption capacity increased from 211.1 to 315.1 mg/g with increased temperature from 25 to 65 °C, indicating that a high adsorption temperature facilitates adsorption. This may be due to raising the temperature, possibly leading to a swelling effect within the hierarchical inter-nanostructure of the Ln@AC, which is conducive to Zn(II) penetrating into the interlayer space and chemically bonding with Ln@AC. It was found that higher temperature was advantageous for adsorption, and adsorption is an endothermic and spontaneous process [24]. In the following experiments, an adsorption temperature of 65 °C was chosen as the ideal condition.

#### 3.2.6. Effect of Adsorption Time

The effects of different adsorption times on the adsorption capacity of Zn(II) by Ln@AC are illustrated in Figure 5f. It can be clearly derived that the trend of the adsorption capacity of the Ln@AC toward Zn(II) increased rapidly in the initial stages, then gradually with prolonged adsorption time until equilibrium was reached. A maximum adsorption capacity of Zn(II) on the Ln@AC (315.0 mg/g) was observed at 50 min. This may be considered as a result of Zn(II) being introduced to the Ln@AC surface for a short contact time, followed by spreading into the hierarchical interlayers, and eventually forming a complex with the active sites and groups. Therefore, under experimental conditions, 50 min adsorption time was selected to ensure that equilibrium was reached.

### 3.3. Adsorption Kinetics

Adsorption kinetics are important for the practicality of the process as they can provide valuable insight into the rate of Zn(II) uptake. Several adsorption kinetic models have been developed and employed to analyze experimental data, identify the adsorption mechanism, and determine the potential rate-controlling steps by the Ln@AC. In this work, four kinetic models were applied to distinguish the model that represented the best fit of adsorption equilibrium data [25,26,27,28].

#### 3.3.1. Pseudo-First-Order Model

The pseudo-first-order adsorption model has been widely used to simulate sorption kinetics data, and it can be expressed as Equation (4):

(4) $$\log(q_{e}-q_{t})=\log q_{e}-\frac{k_{1}t}{2.303}$$

where *q_e_* and *q_t_* denote the adsorbed amounts (mg/g) at equilibrium and time *t* (min), respectively, and *k*_1_ (min^−1^) is the pseudo-first-order rate constant.

#### 3.3.2. Pseudo-Second-Order Model

The pseudo-second-order kinetic model was found to explain most adsorption systems well. This model is shown by Equation (5):

(5) $$\frac{t}{q_{t}}=\frac{1}{k2q_{e}{}^{2}}+\frac{t}{q_{e}}$$

where *q_e_* and *q_t_* are the amounts of Zn(II) adsorbed (mg/g) at equilibrium and time *t* (min), respectively, and *k*_2_ (g (mg/min)^−1^) is the constant rate of the pseudo-second-order kinetic equation for adsorption.

#### 3.3.3. Elovich Kinetic Model

The Elovich kinetic model assumes that the adsorption sites are heterogeneous and display a variety of activation energies during the adsorption process. This model is expressed by Equation (6):

(6) $$q_{t}=\frac{1}{\beta }\ln(\alpha \beta )+\frac{1}{\beta }\ln t$$

where *q_t_* is the amount of adsorption (mg/g) at time *t* (min); *α* (mg·(g·min)^−1^) is an initial adsorption rate; and *β* (g/mg) is related to surface coverage and activation energy for chemisorption.

#### 3.3.4. Intraparticle Diffusion Model

The adsorption process comprises two sequential steps: (a) transporting solution molecules from the aqueous phase to the solid surface; and (b) diffusing the solute molecules into the interior of the structure. The intraparticle diffusion model describes the rate-determining step of the adsorption diffusion process. This model is represented by Equation (7):

(7) $$q_{t}=k_{i}t^{0.5}$$

where *q_t_* is the amount of adsorption (mg/g) at time *t* (min), and *k_i_* (mg·(g·min^0.5^)^−1^) is an intraparticle diffusion rate constant.

**Figure 6:**
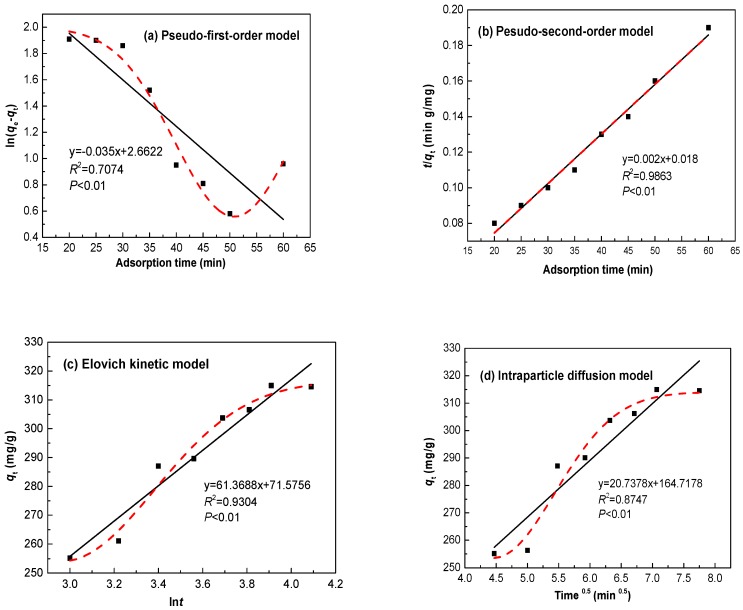
(**a**) Pseudo-first-order, (**b**) pseudo-second-order, (**c**) Elovich kinetic, and (**d**) intraparticle diffusion models for the adsorption of Zn(II) by the Ln@AC nanocomposite.

The Zn(II) adsorption experimental rate data were fitted to the four kinetic models. All kinetics data for adsorption onto the Ln@AC nanocomposite calculated from the related linear and nonlinear fitting curves (Figure 6) are shown in Table 2. The correlation coefficient *R*^2^ was used to compare the fitting quality of the kinetic curves. Figure 6a shows a plot of ln(*q_e_* − *q_t_*) as a function of time, according to the linearized pseudo-first-order model. The low value of *R*^2^ (0.7074) shows that the adsorption of Zn(II) does not follow the pesudo-first-order model. Similarly, Figure 6b presents the plot of (*t*/*q_t_*) versus *t* in the linearized pesudo-second-order model. The magnitude of adsorption capacity (318.7 mg/g), according to this model, was found to be very close to the experimental *q*_max_ value (315.9 mg/g), and *R*^2^ was found to be 0.9863, which suggests the pesudo-second-order model fitted the adsorption process well. In Figure 6c, *q*_t_ was plotted versus ln*t* according to the Elovich kinetics model. *R*^2^ was 0.9304, indicating that the adsorption sites were heterogeneous and a variety of activation energies occurred during the adsorption process; that is to say, the adsorption was also applied with the Elovich model. The fit of the intraparticle diffusion model was determined by the linear plot of *q_t_* versus *t*^0.5^ (Figure 6d). *R*^2^ was found to be 0.8747, implying that the adsorption did not follow this model. A comparison of the *R*^2^ values vividly shows that the adsorption kinetics followed the pesudo-second-order model, as it exhibited the highest *R*^2^; moreover, the Elovich model was also described, and the results suggest that the adsorption rate of Zn(II) on the Ln@AC was mainly controlled by a chemical adsorption process.

### 3.4. Adsorption Isotherms

In order to understand the mechanism of Zn(II) adsorption on the Ln@AC, the Langmuir, Freundlich, Temkin, and Dubinin–Radushkevich isotherm models were used to evaluate the experimental data obtained from the Zn(II) adsorption experiments in this study [29,30,31].

#### 3.4.1. Langmuir Isotherm Model

The Langmuir isotherm model provides a description of monolayer adsorption onto a surface that is not followed by further adsorption. There is no transmigration of the adsorbate in the plane of the surface and adsorption energies are uniform on the surface. The Langmuir isotherm can be expressed as Equation (8):

(8) $$\frac{C_{e}}{q_{e}}=\frac{1}{bq_{m}}+\frac{C_{e}}{q_{m}}$$

where *C_e_* is the concentration of Zn(II) at equilibrium (mg/L); *q_e_* is the amount adsorbed (mg/g); *q_m_* is the complete monolayer adsorption capacity (mg/g); and *b* is the Langmuir constant related to the adsorption capacity (L/mg).

Further analysis of the Langmuir isotherm can be undertaken on the basis of a dimensionless equilibrium parameter (*R*_L_), expressed as Equation (9):

(9) $$R_{L}=\frac{1}{1+K_{L}C_{0}}$$

where *K*_L_ (L/mg) is the Langmuir adsorption constant and *C*_0_ is the initial concentration of Zn(II) ions (mg/L). The value of *R*_L_ indicates the nature of the isotherm as unfavorable (*R*_L_ > 1), linear (*R*_L_ = 1), favorable (0 < *R*_L_ < 1), or irreversible (*R*_L_ = 0).

#### 3.4.2. Freundlich Isotherm Model

The Freundlich isotherm model is an empirical equation that assumes the presence of multilayered heterogeneous adsorption energies on the adsorption surface with possible interaction between adsorbed metal ions. The linear form is given by Equation (10):

(10) $$\ln q_{e}=\ln k_{f}+\frac{1}{n}\ln C_{e}$$

where *C_e_* is the concentration of Zn(II) at equilibrium (mg/L); *q_e_* is the amount adsorbed (mg/g); *k_f_* represents the adsorption capacity when Zn(II) equilibrium concentration equals 1 (mg^(1−1/n)^ L^(1/n)^ g); and *n* is the degree of dependence of adsorption with equilibrium concentration.

#### 3.4.3. Temkin Isotherm Model

The Temkin isotherm model suggests a linear decrease of adsorption energy as the degree of completion of the sorption centers of an adsorbent increase, and the heat of adsorption of all molecules in the interlayer would decrease linearly with coverage due to solid–liquid interactions. The Temkin isotherm can be expressed in the following linear form as Equation (11):

(11) $$q_{e}=\frac{RT}{b_{t}}\ln\alpha_{t}+\frac{RT}{b_{t}}\ln C_{e}$$

where *C_e_* is the concentration of Zn(II) at equilibrium (mg/L); *q_e_* is the adsorption capacity at equilibrium (mg/g); *R* is the ideal gas constant (8.314 J mol^−1^ K^−1^); *T* is the absolute temperature of the adsorption process (K); *α_t_* is the equilibrium binding constant (L/mg); and *b_t_* is related to the heat of adsorption (J/mol).

#### 3.4.4. Dubinin–Radushkevich Isotherm Model

The Dubinin–Radushkevich (D–R) isotherm model was originally used to describe the adsorption of subcritical vapors onto microporous solids following a pore filling mechanism. It can express adsorption onto heterogeneous surfaces with a Gaussian energy distribution. The D–R model allows the computing of mean free energy, which is the energy required to remove a molecule from its location in adsorption space to infinity. The value of mean free energy is often considered a good indicator to distinguish between the chemical and physical adsorption of metals. The D–R model and free energy can be represented by the following expressions (Equations (12) and (13)):

(12) $$\ln q_{e}=\ln q_{\max}-B\varepsilon^{2}$$

(13) $$\varepsilon =RT\ln(1+\frac{1}{C_{e}})$$

where *q*_max_ is the monolayer saturation adsorption capacity (mg/g); *C_e_* is the concentration of metal ions at equilibrium (mg/L); *q_e_* is the adsorption capacity at equilibrium (mg/g); *R* is the ideal gas constant (8.314 J mol^−1^ K^−1^); *T* is the absolute temperature of the adsorption process (K); *B* is a D–R constant (mol^2^ kJ^−2^), and *ε* is the Polanyi potential (kJ/mol).

**Figure 7:**
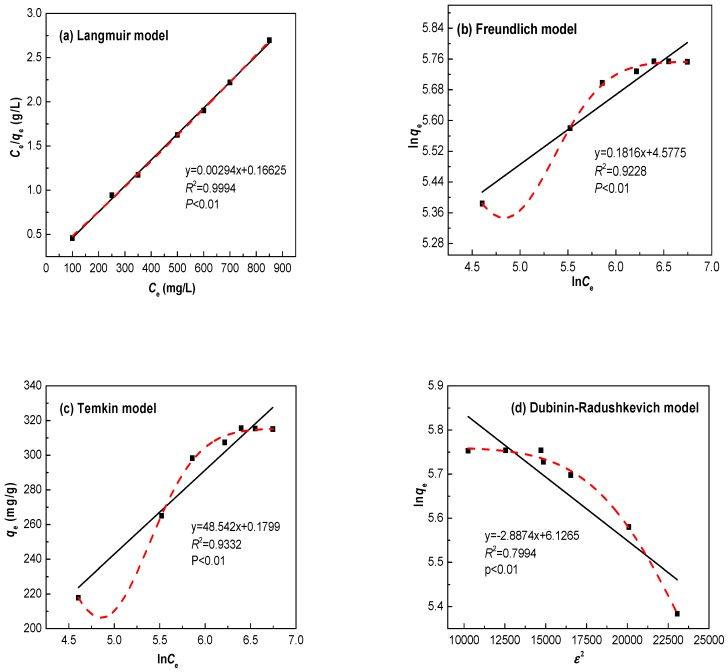
(**a**) Langmuir, (**b**) Freundlich, (**c**) Temkin, and (**d**) Dubinin–Radushkevich isotherm models for adsorption of Zn(II) by the Ln@AC nanocomposite.

The adsorption experimental data for Zn(II) onto the Ln@AC nanocomposite were analyzed by the Langmuir, Freundlich, Temkin, and Dubinin–Radushkevich isotherm models using the linear and nonlinear regression fitting curves to identify the parameters that give the best fit between a set of data and the proposed plots. The fitting plots of the experimental data for the four isotherms are displayed in Figure 7, and the isotherm parameters are listed in Table 3. From Figure 7a and Table 3, it is clear that the Langmuir plot had good linearity, since the correlation coefficient *R*^2^ value was 0.9994, which was closer to 1 than the values of the other models. The maximum monolayer adsorption capacity (*q*_max_) value calculated from the Langmuir model was 313.8 mg/g, which is almost the same as that in the experimental data (315.9 mg/g). The value of *R*_L_ for the adsorption of Zn(II) on the Ln@AC nanocomposite was 0.134, between 0 and 1, confirming that the Ln@AC was favorable for Zn(II) adsorption under the employed adsorption conditions. The *R*^2^ of the Freundlich plot (Figure 7b) was relatively lower when compared to the Langmuir plot. The value of 1*/n* indicated the favorability of the adsorption of Zn(II). The Temkin isotherm constant *b*_t_ was 37.08 J/mol, indicating the physicochemical nature of the adsorption process (Figure 7c). Based on the *R*^2^ value (0.9332), the Temkin model did not perfectly fit the data. The D–R isotherm model was applied to determine the apparent free energy of adsorption ε, which would provide insight on the adsorption mechanism. If the value of ε is <8 kJ/mol, the process is dominated by physisorption, and if it lies in the range of 8–20 kJ/mol, the adsorption is dominated by a chemical bonding process. From Figure 7d and Table 3, it can be seen that the obtained mean free energy ε was 14.50 kJ/mol, illustrating that the adsorption process was characteristic chemical adsorption [32]. Obviously, the Langmuir model describes the adsorption of Zn(II) ions onto the Ln@AC nanocomposite much better than the other models. This demonstrates that the monolayer coverage of Zn(II) was formed on the surface of the Ln@AC nanocomposite. The *q*_max_ value of the Ln@AC nanocomposite was compared with that of different adsorbents (Table 4). It can be seen that the *q*_max_ value of other adsorbents was much lower than that of the Ln@AC nanocomposite. Consequently, this novel adsorbent has excellent adsorption performance and could be useful in treating Zn(II) wastewater.

### 3.5. Desorption and Regeneration

**Figure 8:**
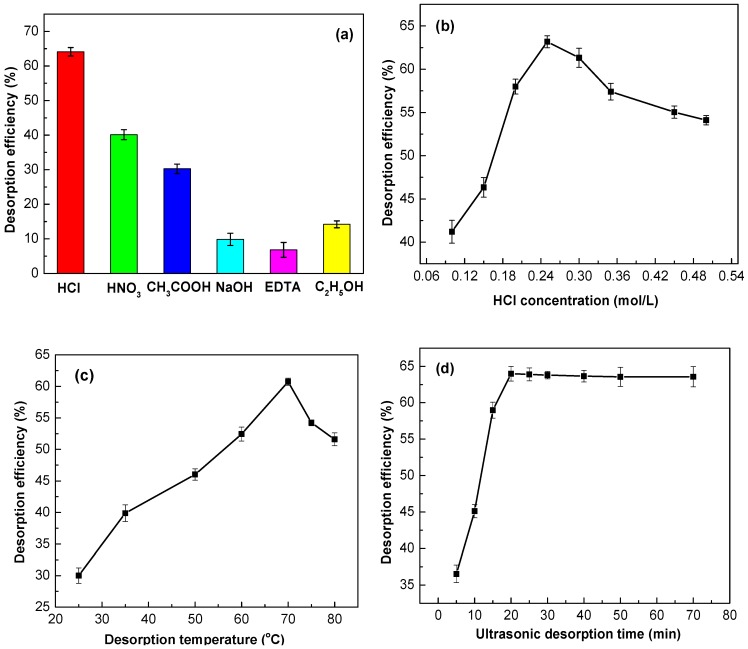
Various influencing factors on the Zn(II) desorption by the Ln@AC nanocomposite. (**a**) Effect of desorbed agents (dosage: 1 g/L; concentration: 0.25 mol/L; desorption temperature: 70 °C; time: 20 min). (**b**) Effect of HCl concentration (dosage: 1 g/L; HCl concentration: 0.10–0.50 mol/L; desorption temperature: 70 °C; time: 20 min). (**c**) Effect of desorption temperature (dosage: 1 g/L; HCl concentration: 0.25 mol/L; desorption temperature: 25–80 °C; time: 20 min). (**d**) Effect of ultrasonic desorption time (dosage: 1 g/L; HCl concentration: 0.25 mol/L; desorption temperature: 70 °C; time: 5–70 min).

The regeneration of the spent Ln@AC nanocomposite for successive removal of Zn(II) from aqueous solution is very important for technical applications. The recovery of Zn(II) from the Ln@AC was studied using different eluting solutions or stripping agents to test their effects on desorption performance. Batches of desorption experiments were conducted in the present work using HCl, HNO_3_, CH_3_COOH, EDTA (ethylene diamine tetraacetic acid), C_2_H_5_OH, and NaOH as the desorbing eluent to test their effects on desorption (Figure 8a): 0.25 mol/L HCl, 0.25 mol/L HNO_3_, 0.25 mol/L CH_3_COOH, 0.25 mol/L EDTA, 0.25 mol/L C_2_H_5_OH, and 0.25 mol/L NaOH. From Figure 8a, it can be clearly seen that EDTA (6.7%) is almost useless for desorbing bonded Zn(II). The desorption efficiency of C_2_H_5_OH was slightly higher than NaOH, but still displayed a lower value when compared with the acid solutions. Among the three acidic desorption eluents (HCl, HNO_3_, and CH_3_COOH), HCl was found to be the best eluent for Zn(II)-loaded Ln@AC. This result reveals that Zn(II) ions on the surface of the adsorbent are more easily exchanged with a large amount of H^+^ existing in the solution, and electrostatic interactions occurred between H^+^ and the active groups, leading to the desorption of positively charged Zn(II). Furthermore, ion exchange, electrostatic attraction, coordination, and chelation, etc., were involved in the adsorption mechanism, and HCl could be an effective desorbing agent for the regeneration of Zn(II)-loaded Ln@AC.

The effect of using HCl solution as a desorbing reagent on the desorption efficiency of Zn(II)-loaded Ln@AC is presented in Figure 8b. As the figure shows, the desorption efficiency initially rapidly increased and then decreased with increasing HCl concentration, possibly because the accumulated H^+^ concentration was the driving force of desorption of Zn(II), to a degree, by positive ion exchange and increased the concentration gradients of Zn(II) and H^+^, further facilitating the desorption. However, when HCl concentration above 0.25 mol/L, the desorption efficiency of Zn(II) decreased, this behavior may be due to the strong electrostatic repulsion between Zn(II) and a sharp increased H^+^ positively charged, which exist in the solution and on the surface of the Ln@AC, causing an effective restriction on the Zn(II) desorption process. The relatively high desorption efficiency (63.17%) at an HCl concentration of 0.25 mol/L suggests that adsorption of Zn(II) onto the Ln@AC was carried out partially via chemical electrostatic attraction and ion exchange, which substantiates the results of the pH value (Section 3.2.4) with respect to the adsorption process.

The effect of desorption temperature on desorption efficiency is shown in Figure 8c. As seen from Figure 8c, the desorption efficiency of Zn(II) increased with increasing temperature, but the desorption efficiency significantly decreased with increasing temperature when the desorption temperature exceeded 70 °C. This phenomenon may be due to increasing temperature, enhancing the activity of adsorption sites on the surface of Ln@AC. H^+^ and loaded Zn(II) may compete with each other for activation sites, leading to increased desorption efficiency [42]. Furthermore, the adsorption process includes physical and chemical adsorption. When the desorption temperature was below 65–70 °C, physical adsorption played a dominant role, which is a fast and reversible equilibrium process, so that both the adsorption and desorption rates increased continuously. As the desorption temperature rose, chemical adsorption is the main process, then the number of activated molecules increased, and the desorption efficiency also increased. When the temperature reached 70 °C, the desorption efficiency reached the maximum. Nevertheless, higher temperature (>70 °C) may have a detrimental effect on the desorption process, thus continued temperature increase was found to have a declining influence on desorption efficiency, which further supports the results of adsorption temperature.

Figure 8d illustrates the effect of ultrasonic desorption time on Zn(II) adsorption. As can be seen, desorption efficiency increased during the first stage, then remained almost constant with increasing ultrasonic desorption time. The results may be due to the ultrasound rules of producing holes; afterward, the reduction is a result of the formation of hydroxyl radicals under ultrasonic conditions by sonication cavitation. This process includes formation, growth, and collapse by violent implosions to release pressure at local hot spots in the aqueous solution. In addition, electrostatic attraction, ion exchange, coordination, and chelation, and so on also exist during the sonication desorption process. Comparatively, a high desorption efficiency of Zn(II) was reached at an ultrasound desorption time of 20 min.

Subsequently, the synthesized Ln@AC nanocomposite was assessed for deterioration by subjecting it to repeated adsorption/desorption experiments, and the results are summarized in Table 5. The experimental data revealed that the Ln@AC nanocomposite exhibited a certain recyclable regeneration performance [43].

### 3.6. SEM/EDX (Scanning Electron Microscopy/Energy Dispersive X-Ray Spectroscopy) Analysis

**Figure 9:**
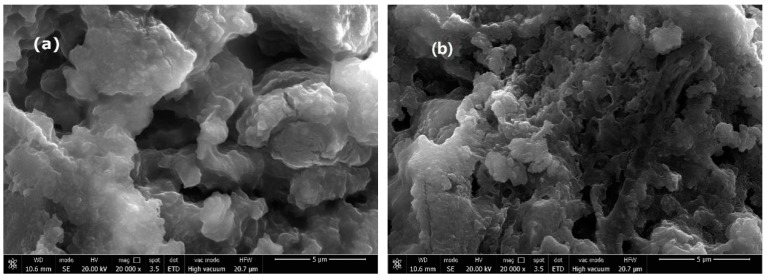
The SEM (scanning electron microscopy) images of the Ln@AC nanocomposite (**a**) before and (**b**) after adsorption of Zn(II).

The SEM analysis depicts the surface morphology changes of unloaded and metal-loaded adsorbents. The SEM images of Ln@AC nanocomposite before and after Zn(II) adsorption are shown in Figure 9. From Figure 9a, it can be clearly seen that the surface of the Ln@AC consisted of many thin layers, small sheets, numerous cavities, and a porous surface with irregular shapes, steps, and broken edges, which may be an appropriate hierarchical interstructure for Zn(II) adsorption [44]. After adsorption of Zn(II), the Ln@AC surface was evenly packed with Zn(II) particles and the sheet-stacking structure disappeared (Figure 9b). The hierarchical nanostructure became smoother with less porosity; most probably, Zn(II) was entrapped and adsorbed on Ln@AC, hinting that Zn(II) was mainly retained in the microporous surface and interlayer space and only selected functional groups were involved in the adsorption. Overall, it indicates that the adsorption of Zn(II) may be a chemical interaction, supporting the previously proposed adsorption mechanism.

**Figure 10:**
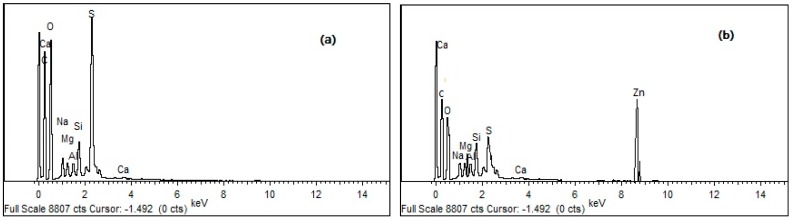
EDX (energy dispersive X-ray spectroscopy) analysis of Ln@AC nanocomposite (**a**) before and (**b**) after adsorption of Zn(II).

The EDX analysis of the Ln@AC was performed to confirm the presence of Zn(II) on Zn(II)-loaded Ln@AC. Figure 10 presents the EDX spectra of pure Ln@AC (Figure 10a) and Zn(II)-loaded Ln@AC (Figure 10b). In the EDX spectrum of Zn(II)-loaded Ln@AC (Figure 10b), a new peak of Zn(II) was found, verifying the presence of Zn(II) ions. The elemental composition of Ln@AC before and after Zn(II) adsorption was analyzed. The EDX results of the purified Ln@AC show the presence of C (69.82%), O (12.11%), Ca (8.23%), S (5.29%), Na (0.15%), Si (2.19%), Mg (0.87%), and Al (1.34%). After Zn(II) adsorption, the *at %* values of Zn(II)-loaded Ln@AC were 67.35% C, 8.87% O, 5.96% Ca, 4.13% S, 0.11% Na, 0.97% Si, 0.66% Mg, 0.90% Al, and 11.05% Zn. This result verifies the presence of Zn(II) on the surface of the Ln@AC after adsorption. From the SEM/EDX results, it was concluded that the Ln@AC nanocomposite had satisfactory performance with highly efficient adsorption capacity for Zn(II) removal from aqueous solutions.

## 4. Conclusions

A novel Ln@AC nanocomposite with a hierarchical nanostructure was successfully prepared by intercalation between Ln and AC and employed for the removal of Zn(II) from an aqueous solution. The experimental results show that Ln@AC had superior adsorption capacity for Zn(II), which can be attributed to its high surface area (611.23 m^2^/g), total pore volume (3.654 cm^3^/g), negatively charged zeta potential (–30.17 mV), and high presence of O-containing functional groups to adsorb Zn(II) ions. Batches of adsorption tests were conducted, and the experimental values obtained were found to be in good agreement with those predicted by the models. The adsorption process was well fitted with the pseudo-second-order kinetic model, the Langmuir isotherm model, and the Elovich model were both suitable for the adsorption equilibrium data of Zn(II) on the surface of the Ln@AC nanocomposite. The maximum desorption capacity and efficiency of the Ln@AC washed with HCl were 202.5 mg/g and 64.10%, respectively. Furthermore, the SEM/EDX results demonstrated that Zn(II) was mainly adsorbed by selected activated groups in coarse mesopores on the surface of the Ln@AC nanocomposite, and the adsorption mechanism was likely a chemisorption process. Hence, the Ln@AC nanocomposite was confirmed as being a highly efficient adsorbent to remove Zn(II) from an aqueous solution.

## Figures and Tables

**Table 1:** Pore structure parameters and zeta potential of lignocellulose@ activated clay (Ln@AC) studied in this work.

Sample	S*_BET_* (m^2^/g)	S*_ext_* (m^2^/g)	S*_ext_*/S*_BET_* (%)	V*_tot_* (cm^3^/g)	V*_meso_* (cm^3^/g)	V*_mic_* (cm^3^/g)	V*_m__ac_* (cm^3^/g)	V*_meso_*/V*_tot_* (%)	D*_p_* (nm)	Zeta Potential (mV)
AC	279.14	98.81	35.40	0.944	0.325	0.152	0.448	34.43	80.23	−17.69
Ln@AC	611.23	489.07	80.01	3.645	2.047	0.566	0.981	56.16	54.09	−30.17

Number of analyses: three. S*_BET_*, specific surface area; S*_ext_*, mesopore surface area; S*_ext_*/S*_BET_*, ratio of mesopore surface area to specific surface area; V*_tot_*, total pore volume; V*_meso_*, mesopore volume; V*_m__ic_*, micropore volume; V*_m__ac_*, macropore volume; V*_meso_*/V*_tot_*, ratio of mesopore volume to total pore volume; D*_p_*, average pore size.

**Table 2:** *R*^2^ and constant values for the adsorption kinetics models of Zn(II). Adsorption experiments: weight ratio of Ln to AC: 3:1; dosage: 1 g/L; initial Zn(II) concentration: 600 mg/L; pH: 6.8; adsorption temperature: 65 °C; adsorption time range: 20–60 min.

Metal	Parameter	Pseudo-First-Order		Pseudo-Second-Order		Elovich Model		Intraparticle Diffusion	
Zn(II)	*R* ^2^	0.7074		0.9863		0.9304		0.8747	
	Constants	*k_1_*	0.0066 min^−1^	*k_2_*	0.0810 g(mg/min)^−1^	*α*	22.06 mg/(g min)	k_i_	8.053 mg/(g min^0.5^)
		*q_e_*	194.3 mg/g	*q_e_*	318.7 mg/g	*β*	0.051 g/mg		

**Table 3:** The *R* ^2^and constant values for the adsorption isotherm models of Zn(II). Adsorption experiments: weight ratio of Ln to AC: 3:1; dosage: 1 g/L; initial Zn(II) concentration: 100–850 mg/L; pH: 6.8; adsorption temperature: 65 °C; adsorption time: 50 min.

Metal	Parameter	Langmuir		Freundlich		Temkin		Dubinin–Radushkevich	
Zn(II)	*R* ^2^	0.9994		0.9228		0.9332		0.7994	
	Constants	*K_L_*	0.022 L/mg	*K_f_*	84.05 mg^(1−1/n)^L^(1/n)^g	*b_t_*	37.08 J/mol	*B*	7.052 × 10^−8^ mol^2^ J^2^
		*R_L_*	0.134						
								*ε*	14.50 kJ/mol
		*q_max_*	313.8 mg/g	*1/n*	0.41	*a_t_*	4.751×10^8^ L/g	*q_max_*	180.4 g/mg

**Table 4:** The *q*_max_ values for the adsorption of Zn(II) on different adsorbents.

Adsorbent	*q*_max_ (mg/g)	Reference
Ln@AC nanocomposite	315.90	This paper
Graphene oxides	208.33	[33]
Polyethyleneimine crosslinked cellulose/sodium alginate	110.2	[34]
Fe_3_O_4_–Si–COOH	110	[35]
XSBL activated carbon	103.82	[36]
EDTA–silica	74.07	[37]
Fe_3_O_4_ and g–C_3_N_4_	45	[38]
Multicarboxyl-functionalized silica gel	39.96	[39]
PVC-acetylacetone composites	26.65	[40]
Coconut tree sawdust	23.81	[41]

**Table 5:** The Ln@AC nanocomposite adsorption/desorption capacity and desorption efficiency of Zn(II) after multiple cycles.

Recycle Time.	1st	2nd	3rd	4th	5th	6th
Adsorption *q*_e_ (mg/g)	315.9	269.8	211.0	154.3	78.1	60.1
Desorption *q*_e_ (mg/g)	202.5	127.3	84.0	37.1	11.2	4.9
Desorption efficiency (%)	64.10	47.18	39.81	24.04	14.34	8.15
